# Global Vaccine Action Plan Lessons Learned II: Stakeholder Perspectives

**DOI:** 10.1016/j.vaccine.2020.05.048

**Published:** 2020-07-14

**Authors:** Angela Hwang, Chantal Veira, Stefano Malvolti, Thomas Cherian, Noni MacDonald, Christoph Steffen, Ian Jones, Alan Hinman, Carsten Mantel

**Affiliations:** a Angela Hwang Consulting, P.O. Box 6601, Albany, CA, 94706, USA; b Task Force for Global Health, 330 West Ponce de Leon Ave., Decatur, GA, 30030, USA; c MMGH Consulting GmbH, Kuerbergstrasse 1, 8049 Zurich, Switzerland; d Dalhousie University, IWK Health Centre, Halifax, Canada; e Immunization, Vaccines and Biological Department, World Health Organization, Geneva, Switzerland; f Jinja Publishing Ltd, Bishop’s Stortford, United Kingdom

**Keywords:** GVAP, Global Vaccine Action Plan, IA2030, Immunization Agenda 2030, Evaluation, Immunization strategy, CSOs, Civil society organizations, DoV, Decade of Vaccines, GVAP, Global Vaccine Action Plan 2011-2020, IA2030, Immunization Agenda 2030, M&E/A, Monitoring and Evaluation/Accountability, NITAG, National immunization technical advisory group, R&D, Research and Development, SAGE, Strategic Advisory Group of Experts on Immunization, UNICEF, United Nations Children’s Fund, SO, Strategic Objective, WHA, World Health Assembly, WHO, World Health Organization

## Abstract

**Introduction:**

The Global Vaccine Action Plan (GVAP), unanimously endorsed by the World Health Assembly in 2012, defined an ambitious strategy to improve immunization. At the end of the decade, significant progress has been made but four of the five GVAP goals are likely to be missed. This report describes a set of surveys and interviews relating to GVAP, conducted to inform the immunization strategy for the next decade.

**Methods:**

Three surveys and two sets of semi-structured interviews were conducted from 2017 to 2019. Respondents consisted of immunization stakeholders at global, regional, and country levels, and included individuals who had been involved in the development and implementation of GVAP or its monitoring, evaluation and accountability (M&E/A) process; national immunization managers; academics; and personnel from non-governmental organizations and civil society organizations.

**Results:**

The surveys and interviews gave consistent results. They highlighted the value of GVAP in increasing visibility for immunization and the benefits of the GVAP M&E/A framework. The main limitations of GVAP were identified as the limited ownership by countries and other stakeholders leading to incomplete implementation of the strategy and poor accountability for achieving GVAP targets.

**Discussion:**

These results informed the review of GVAP and the development of its successor strategy, the Immunization Agenda 2030. In addition, these surveys and interviews identified two challenges in assessing the value of GVAP: the need to rely exclusively on stakeholder perspectives and difficulties in attributing benefits. These challenges are inherent in evaluating an over-arching strategy such as GVAP and should be factored into interpretation of the results.

## Highlights

• GVAP built visibility for immunization and was an improvement over prior plans.
• GVAP implementation was incomplete due to limited awareness and stakeholder buy-in.
• The GVAP Monitoring & Evaluation/Accountability framework gave valuable guidance.
• Future strategies should build greater buy-in and communicate more effectively.

## 1 Introduction

The Global Vaccine Action Plan 2011–2020 (GVAP) was unanimously endorsed in 2012 by the World Health Assembly (WHA). GVAP aimed to realize the vision of the Decade of Vaccines (DoV), of “a world in which all individuals and communities enjoy lives free of vaccine-preventable diseases.” It defined a global strategy for immunization, including specific goals and strategic objectives (SOs, see Supplemental Materials Appendix A); the actions required to achieve those goals and objectives; and stakeholder responsibilities for implementing the strategy. It established a Monitoring and Evaluation/Accountability (M&E/A) framework to track and drive progress. This framework included annual results measurement and reporting; independent progress assessments by the Strategic Advisory Group of Experts on Immunization (SAGE); SAGE recommendations for corrective actions; and discussions of the assessment report at the WHA [1].

As the end of the decade approaches, it is very likely that four of the five GVAP goals will not be met. Reaching vaccine coverage and equity targets; eradicating polio; and eliminating measles, rubella, congenital rubella syndrome, and maternal and neonatal tetanus remain stubborn challenges. Child mortality has been reduced by 59% from 1990 to 2018, falling short of the Millennium Development Goal target to reduce child mortality by two-thirds by 2015 [2]. However, significant progress has been made in new vaccine introduction and vaccine research and development (R&D) [3].

Anticipating the conclusion of the decade, SAGE called for a comprehensive review of the progress, impact, and implementation of GVAP to inform the development of the next global immunization strategy, the Immunization Agenda 2030 (IA2030) [4]. In response to that call, this paper synthesizes stakeholder feedback on GVAP and highlights its strengths and limitations. The comprehensive review of GVAP by the SAGE DoV Working Group is given in an accompanying article [5].

## 2 Methods

Multiple surveys and sets of stakeholder interviews relating to GVAP have been conducted, as summarized in Table 1 and described further below. All targeted immunization stakeholders at the global, regional, and country levels, and some stakeholders responded to multiple surveys and interviews. Survey 1 was conducted independently. The remaining surveys and interviews were commissioned and overseen by the World Health Organization (WHO). Survey 3 and Interview Series 2 were conducted by the authors of this report. Since these were evaluations of a global strategy for assessment and improvement purposes, ethical review and informed consent were not deemed necessary.

**Table 1:** GVAP Surveys and Interviews.

	**Focus**	**Conducted**	**Responses**				**Response Rate**
			**Total**	**Global**	**Regional**	**Country**	
**Interview Series 1**	Lessons learned from GVAP and key process issues for the 2021–2030 strategic plan [6]	May – June 2017	40	28	11	1	69%
**Survey 1**	Utility and application of GVAP and suggestions for the post-2020 strategy [7]	2017 – 2018	96	38	6	52	Phase 1 - up to 43%^1^ Phase 2 – 87%
**Survey 2**	The “why, what, and how?” of a post-2020 global immunization strategy [8]	June 2018	158	85	24	49	62%
**Survey 3**	Contribution of GVAP to improving global immunization	February – April 2019	56	30	12	14	49%
**Interview Series 2**	Value-added of GVAP	March – May 2019	40	18	8	14	67%

1 Survey recipients were encouraged to forward the link to colleagues. As a result, the total number of survey recipients is unknown and the exact response rate cannot be calculated.

### 2.1 Interview Series 1

Semi-structured interviews were conducted with 40 global, regional and country stakeholders representing 23 organizations. Respondents included national immunization managers, representatives of United Nations agencies, global immunization partners, civil society organizations (CSOs), industry, and academia. A briefing note was shared in advance, describing lessons learned from the process of GVAP development and key issues and needs for the development of the 2021–2030 immunization strategy. Interview questions explored the development of GVAP, the need for a successor to GVAP, and key process considerations for the future immunization strategy. (For the questions see Supplemental Material, Appendix B.) Responses were summarized based on the viewpoints expressed by the majority of responders and strongly dissenting views were highlighted. The results of these interviews were presented to and discussed with the SAGE DoV Working Group and with SAGE in 2017 [6]. Subsequently, notes from these interviews were incorporated in the thematic analysis described below.

### 2.2 Survey 1

A two-phase survey was administered to immunization stakeholders as previously described [7]. In the first phase in 2017, a convenience sample of 88 representatives of global organizations involved in the development of GVAP was surveyed. Thirty-eight responses were received for this survey. In a second phase in 2018, 20 countries were identified that had sizeable populations and that had reported ≥ 5% increases or decreases in coverage with three doses of diphtheria-tetanus-pertussis vaccine from 2010 to 2016. For those 20 countries, national immunization managers and country representatives from WHO and the United Nations Children's Fund (UNICEF) were surveyed. In addition, the six WHO Regional Advisors for Immunization were surveyed in this phase of the study. Questions addressed global progress toward achieving GVAP goals and the severity of challenges for immunization programs, and stakeholders were offered a textbook as an incentive for completing the survey. Responses were received from 18 immunization managers (90% response rate), 34 country representatives (85%), and 6 regional advisors (100%).

### 2.3 Survey 2

At the June 2018 Global Immunization Meeting in Kigali, Rwanda, 255 participants were surveyed on “The Why, What, and How?” of a post-2020 global immunization strategy. Twenty-five countries were represented, primarily from the African and Eastern Mediterranean regions. Survey questions explored the need for a new strategy and the issues to be addressed in that strategy. (For the questions, see Supplemental Materials Appendix C.) Among the 158 respondents, 31% represented a country perspective (65% response rate), 15% represented a regional perspective (42% response rate), and the remaining 54% represented a global perspective (69% response rate) [8].

### 2.4 Survey 3

In total, 115 stakeholders involved in the development and implementation of GVAP, regional vaccine action plans, or national immunization programs and decision making were surveyed using Qualtrics™, an off-the-shelf survey tool. Respondents were given a list of 36 specific actions relating to GVAP and asked to score each one on their contribution to improving global immunization. Options were 3 for “important contribution”, 2 for “moderate contribution”, 1 for “slight contribution”, and 0 for “no contribution”. In addition, respondents scored the GVAP SOs in terms of contribution to improving global immunization, using the same scoring rubric. (For the SOs and questionnaire, see Supplemental Materials, Appendix A and Appendix D.)

Of the 56 responses received, 54% represented global perspectives (46% response rate) and 46% represented regional or country perspectives (52% response rate). Respondents included academics, country representatives, WHO staff, and immunization partners, and represented all six WHO regions. For each action described in the survey, an average score was computed to facilitate comparisons.

### 2.5 Interview Series 2

Semi-structured interviews were conducted with 40 immunization stakeholders. Global participants were selected with input from the WHO Immunization, Vaccines, and Biologicals department and included key stakeholders in the Decade of Vaccines Collaboration, which formulated GVAP; in ongoing GVAP monitoring and evaluation; and in programs relevant to GVAP goals. Regional and country participants included WHO Regional Advisors for Immunization and at least one country focal point from each region. Country focal points included WHO country office staff, national immunization program representatives, and national immunization technical advisory group (NITAG) members. Thirteen countries and all six WHO regions were represented among the interviewees. Eight global participants and 4 regional participants had previously participated in Interview Series 1, and 70% of the interviewees also received Survey 3.

Questions were sent in advance and addressed the strengths and weaknesses of the GVAP development process, the contribution of GVAP to improving immunization, and aspects of GVAP to retain or change moving forward. (Questionnaire text is available in the Supplemental Materials, Appendix E.) Interview notes were analyzed using the Framework Method [9]. Data were coded to organize the input into themes and stratified according to participant perspectives. Themes reflected the diversity of opinion while also indicating which responses were more common and which were minority views. Themes were reviewed by study team members to ensure accuracy.

An initial thematic analysis of Interview Series 2 has been reported elsewhere [5]. To give a more detailed and complete picture, this analysis was expanded to incorporate notes from Interview Series 1 and the small number of free-text responses entered on Survey 3, yielding results consistent with those reported in the initial analysis. (The detailed thematic analysis is given in Supplemental Materials, Appendix G.)

## 3 Results

### 3.1 Consolidated results of all interviews and surveys 1 and 2

This section summarizes the thematic analysis of both sets of interviews, in combination with reported results from Survey 1 and Survey 2.

**Development seen as “top down.”** In these surveys and interviews, respondents commented that the development of GVAP included robust and well-conducted consultations, but only a handful of organizations were involved in drafting the final plan. As a result, GVAP was perceived as not fully reflecting the breadth and richness of the consultations or the advice of various expert working groups. National governments, CSOs, and other implementing bodies, while consulted, had limited involvement at the later stages, resulting in a strategy seen as “top-down” by stakeholders from all perspectives.

**Unifying strategy**. In spite of these limitations, GVAP was seen as a powerful call for action. It aligned stakeholders around a common agenda and with a common language, which contributed to partnership, collaboration, advocacy, and, in some instances, fundraising. Compared to its predecessor, the Global Immunization Vision and Strategy 2006–2015 [10], it had higher visibility, was more comprehensive, and drew in more stakeholders. However, advocacy and communication relating to GVAP declined over the course of the decade, contributing to a lack of awareness and misconceptions about the strategy, particularly among country stakeholders.

**Resources varied**. Experience regarding resource mobilization to implement GVAP varied from country to country, with some respondents noting that GVAP had helped to mobilize domestic resources and others saying that it had not. Among partner organizations, some said GVAP had helped to increase resources dedicated to immunization while others said that GVAP did not influence their budgets. On the whole, there was a mismatch between the ambitious goals adopted by GVAP and the resources dedicated to achieving them both within countries and across partners.

**Implementation varied**. GVAP endorsement triggered the development or revision of Regional Vaccine Action Plans (RVAPs) that adapted GVAP to regional contexts and served as a guide for implementation at the country level. Some countries aligned their national immunization plans with RVAPs and took the lead in implementing the GVAP strategy. In other cases, the top-down development process and misalignments between GVAP aspirations and country realities contributed to countries not assuming responsibility for GVAP to the same degree. Respondents referred to this lack of “country ownership” as one reason for incomplete implementation, and cited limited domestic financing and political support for immunization as evidence of poor ownership. Many partners did not adopt GVAP in its entirety but defined their own priorities. As a result, some GVAP goals and objectives did not receive sufficient support.

**Measurement and Evaluation an important contribution**. Global M&E/A was considered a highlight of GVAP. Data collection for new indicators such as stockouts or vaccine hesitancy helped to draw attention to these issues. The annual SAGE reports and their recommendations helped to highlight important issues such as data quality and the need to serve vulnerable populations. Discussions at WHAs brought these issues to the attention of Ministers of Health, created opportunities to discuss challenges such as vaccine affordability for middle-income countries, and may have contributed to increased political support for immunization. Concerns regarding the global M&E/A process included too many indicators, too great a focus on missed targets, insufficient recognition of progress, and vague or impractical recommendations.

M&E/A was also seen as an important element of the RVAPs, although there was a greater diversity of opinion as expected given regional diversities. Annual RVAP progress reports were reviewed at regional meetings, underscoring the importance of immunization and drawing attention to countries that were falling behind. Some respondents’ views differed, noting that those reports garnered little attention.

Country level respondents gave widely differing feedback on the M&E/A process. Some noted that it built awareness of progress, but others noted that GVAP-related M&E/A was not helpful. Multiple country level respondents were unaware of the annual GVAP reports and their recommendations, however one country respondent said that the GVAP recommendations helped to mobilize support from national decision makers. Some noted that WHA sessions on GVAP were useful and moved the agenda forward, others said that these discussions were superficial and not linked to actions in the country.

Overall, the M&E/A process was seen as a necessary and important element of GVAP. It was a useful process and contributed to accountability for achieving GVAP goals, but did not succeed in establishing widespread, full accountability for meeting GVAP targets.

**Accountability limited**. Accountability was difficult to achieve due to aspirational targets and because stakeholders were not fully aligned on targets such as measles elimination. It was also difficult to achieve accountability because GVAP lacked a unified management structure directing implementation and controlling resources: the GVAP governance structure was seen as weak, with “no teeth.” Some noted greater accountability to RVAP targets and to the “10 Commitments” of the Addis Declaration on Immunization, which was launched to increase political will for immunization in Africa.

**The value of GVAP**. In addition to aligning stakeholders around a common agenda, respondents noted that GVAP highlighted important issues such as coverage, equity, vaccination after infancy, data quality, vaccine supply and stockouts, and vulnerable populations. It strengthened NITAGs, aligned global stakeholders around an R&D agenda, and brought researchers and implementers together.

**Broader impact difficult to attribute**. Some respondents noted that GVAP helped to improve immunization performance. However, the majority were unable to attribute progress made during the decade specifically to GVAP. Attribution of benefits to GVAP is inherently difficult because the strategy reinforced existing goals and initiatives and was implemented in the context of many overlapping interventions, programs, and strategies supporting immunization, such as those of Gavi, the Vaccine Alliance; WHO; and UNICEF.

**Need for a post-2020 global immunization strategy**. There was consensus among respondents that an updated strategy is needed to sustain the benefits of immunization, complete the unfinished business of GVAP, and address emerging challenges. In addition to advancing existing priorities such as coverage and equity, elimination and eradication targets, data quality, vaccine uptake, and operational research, the new strategy should improve demand for immunization and address vaccine hesitancy, improve access to sufficient supplies of affordable vaccines for middle-income countries, promote the integration of immunization with other health services, and address the needs of people affected by fragility, conflict, and vulnerability.

**Success factors**. Respondents noted that the post-2020 global immunization strategy should be owned by countries, and made several suggestions for increasing country ownership: it should be developed using a bottom-up approach with a greater focus on implementation. It should have a health systems perspective, be integrated into the broader development agenda, and partner with related initiatives and concerns. It should be able to adapt to changing contexts and challenges over the next decade. It should include plans for addressing financial needs at the global, regional, and country levels. The governance mechanism should include country and regional engagement and hold partners accountable for their role in the new strategy. There should be fewer indicators in the new monitoring and evaluation framework. It should monitor progress in implementation as well as outcomes, including implementation of recommendations issued over the course of the decade. Evaluations should factor in country situations, recognize progress even when targets are not met, and explore the root causes for missed targets. Advocacy should be an important component of the new strategy, serving to highlight the importance of immunization and to build political will.

### 3.2 Results from Survey 3

**Fig. 1:**
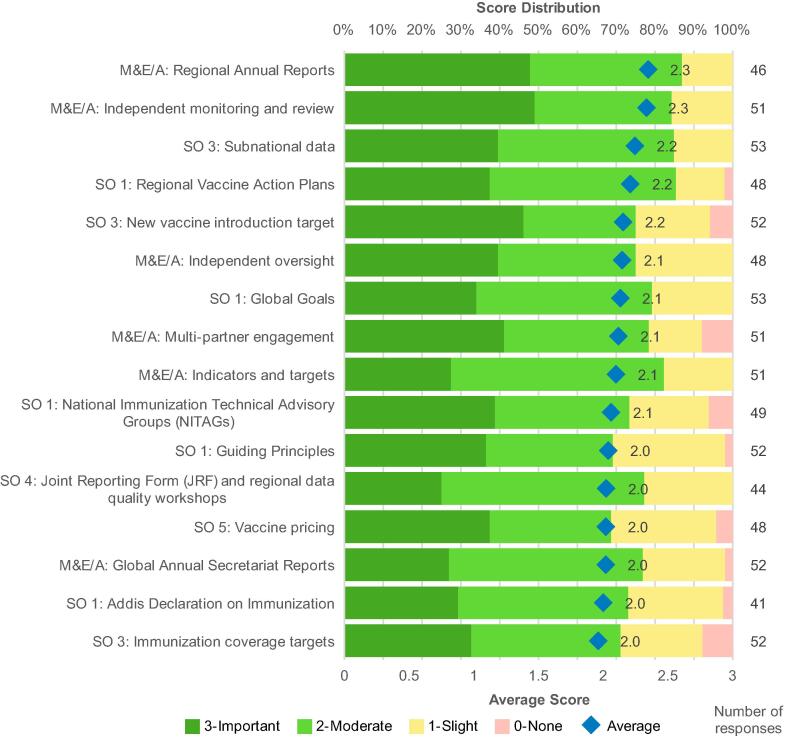
GVAP Contribution to improving global immunization. Perceived GVAP contribution to improving global immunization, actions with average score of 2 or greater, sorted by average score (all respondents combined).

This survey went beyond previous studies to provide greater detail on the benefits of GVAP. Perceptions of the utility of GVAP were positive overall: average survey scores for all 36 GVAP-related actions ranged from 1.4 to 2.3 on a scale from 0 to 3, reflecting moderate contributions to improving immunization (Fig. 1). In general, respondents representing regional and national perspectives gave similar or slightly higher scores than those representing global perspectives (Supplemental Materials, Appendix F).

A comparison of scores across the 36 actions showed broad recognition of the value of the GVAP M&E/A framework and of related measurement and evaluation conducted at the regional level. Of these actions, regional annual reports and the independent monitoring and review process were considered the most impactful in improving accountability. Lessons learned from the GVAP M&E/A framework are discussed in greater detail in the accompanying article [13].

Also recognized were GVAP contributions to building political will for immunization through RVAPs, global goals, NITAGs, guiding principles, and the Addis Declaration on Immunization signed at the Ministerial Conference on Immunization in Africa [11]; and to equity through a focus on subnational data (for improving equity within countries) and access to new vaccines (relating to equity between countries).

**Fig. 2:**
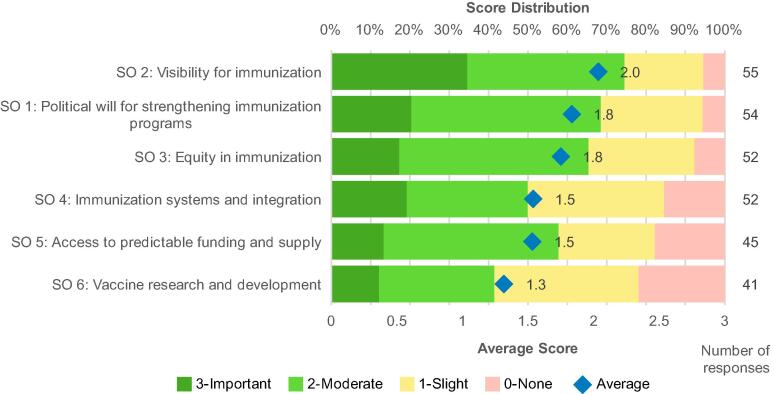
GVAP Contribution to achieving Strategic Objectives. Perceived GVAP contribution to achieving SOs, sorted by average score (all respondents combined).

The six GVAP Strategic Objectives (SOs) are shown in Supplemental Materials Appendix A. When respondents were asked to score the contribution of GVAP to meeting each SO, the SOs received average scores ranging from 1.3 to 2.0, indicating that GVAP had made slight to moderate contributions to achieving each one (Fig. 2). SO 2, Visibility for immunization, SO 1, Political will for strengthening immunization programs, and SO 3, Equity in immunization, were seen as benefitting the most from GVAP.

Respondents were generally more positive in Survey 3, where they were asked to score the contribution of specific actions, than in interviews where they reflected on overall progress. For example, a majority of Survey 3 respondents noted that the M&E/A related actions made important or moderate contributions to accountability for achieving GVAP targets. In contrast, interview respondents generally commented on poor accountability. This difference is likely due to the multifactorial nature of progress, where many activities can contribute to an improvement while still not achieving the desired change.

## 4 Discussion

### 4.1 Challenges in estimating the value of GVAP

Two challenges are faced when assessing the value of an over-arching strategy such as GVAP: the need to rely exclusively on stakeholder perspectives and difficulties in attributing benefits.

First, due to the lack of objective data reflecting the value of GVAP, these conclusions are based on stakeholder surveys and interviews, which have several limitations. As is common in qualitative research, stakeholder responses are subject to social desirability bias. This is an especially important concern in these reviews because respondents were selected from a highly interconnected community of immunization experts. Stakeholder responses also reflected divergent viewpoints on many questions. This was expected, as the investigators deliberately sought a diversity of perspectives. However, because of the breadth of GVAP, divergent viewpoints may also reflect the limitations of individual awareness: although experts, respondents are not likely to be fully cognizant of what is happening in areas outside their specialty. As a result, analysis of their input must take their roles into consideration: this process is subject to confirmation bias and difficult to do while maintaining anonymity.

Second, respondents found it difficult to directly attribute progress to GVAP. For example, GVAP R&D objectives were relatively successful during this decade and two stakeholders noted that GVAP contributed to the engagement of pharmaceutical companies. However, a majority of stakeholders responding to Survey 3 believed that GVAP made slight or no contribution to achieving GVAP R&D objectives (Fig. 2) and several interview respondents considered that the progress in R&D would have occurred in the absence of GVAP, driven by existing R&D programs and strategies. This attribution challenge is inherent in over-arching, “umbrella” strategies such as GVAP that work through the strategies of disease-specific initiatives and stakeholder organizations. In this context, any attribution of benefit will be highly subjective and difficult to validate. In spite of this difficulty, there remains broad acknowledgement of the value of GVAP and a consensus that the strategy should be renewed and strengthened for the next decade.

### 4.2 Limitations

These surveys and interviews had additional limitations that can and should be addressed in future reviews. While having multiple teams evaluating from multiple perspectives can help to establish the reproducibility of the results, in this instance some stakeholders contributed to more than one study, potentially leading to over-representation of certain individual or organizational perspectives. Future interviews and surveys should maintain balanced representation of perspectives, including while synthesizing results across studies. While Survey 2 was translated into a number of languages, due to time and budgetary constraints the remaining surveys and interviews were conducted in English, except for a small number of interviews that were conducted in French. This created communication challenges for some respondents and is likely to have limited responses from non-English speakers. Future studies should ensure balanced representation across language groups. Response rates in the surveys were less than 50% in some cases. While there were no clear trends in responsiveness among global, regional, and country stakeholders, there remains a potential for non-response bias. Future studies should strive to maximize response rates. Stakeholder statements were not fact-checked: some triangulation would have assisted in interpreting their input and reduced the potential for confirmation bias.

## 5 Conclusions

These stakeholder perspectives remain valuable in informing future immunization strategies. Although conducted at different times and with different specific aims, these surveys and interviews gave similar results, showing broad consensus about the strengths and weaknesses of the strategy. The main conclusions were:

• GVAP served to increase visibility for immunization and to align a wider range of stakeholders around a common strategy.
• Implementation of GVAP was incomplete, due to limited country ownership and partner buy-in and due to goals and targets perceived as too ambitious to reach by 2020.
• While many GVAP-related activities contributed to improving immunization, it is difficult to attribute progress specifically to GVAP.
• GVAP M&E/A highlighted the importance of immunization and drew attention to emerging issues but was not sufficient to build accountability for meeting GVAP targets.
• A post-2020 immunization strategy is needed to complete the unfinished business of GVAP, address emerging challenges, and sustain the benefits of immunization.
• Future immunization strategies should build greater buy-in and accountability and communicate more effectively throughout their life cycle.

Detailed reports of these results have been presented to SAGE and are informing the development of the successor strategy, IA2030, as discussed in the accompanying article [5]. In particular, these results have helped to shape the four core principles of IA2030, “People-focused, Country-owned, Partnership-based, and Data-enabled,” which will be applied systematically across the entire strategy [12].

## Disclaimer

The authors alone are responsible for the views expressed in this article and they do not necessarily represent the views, decisions or policies of the institutions with which they are affiliated.

## Declaration of Competing Interest

The authors declare the following financial interests/personal relationships which may be considered as potential competing interests: Angela Hwang served on the DoV M&E/A secretariat from 2013 to 2017 as an employee of the Bill & Melinda Gates Foundation. Thomas Cherian assisted the DoV Collaboration Delivery Working Group and served on the DoV M&E/A Secretariat from 2012 to 2017 as an employee of the WHO. Noni MacDonald has been a member of SAGE and the Chair of the SAGE DoV Working Group since 2017. Christoph Steffen has managed the DoV M&E/A Secretariat since 2017 as an employee of the WHO. Alan Hinman served on the SAGE DoV Working Group from 2013 to 2015 as an independent expert. Carsten Mantel assisted the DoV Collaboration Delivery Working Group as an employee of the WHO. All authors attest they meet the ICMJE criteria for authorship.

## References

[1] World Health Organization. Global Vaccine Action Plan 2011-2020. WHO Press, 2013. http://www.who.int/immunization/global_vaccine_action_plan/GVAP_doc_2011_2020/en/ [accessed February 14, 2019].

[2] UN Inter-agency Group for Child Mortality Estimation. Levels & Trends in Child Mortality Report 2019. United Nations Children's Fund, 2019. https://childmortality.org/wp-content/uploads/2019/10/UN-IGME-Child-Mortality-Report-2019.pdf [accessed December 4, 2019].

[3] World Health Organization Strategic Advisory Group of Experts on Immunization. The Global Vaccine Action Plan 2011-2020 Review and Lessons Learned. 2019. https://apps.who.int/iris/bitstream/handle/10665/329097/WHO-IVB-19.07-eng.pdf [accessed November 19, 2019].

[4] World Health Organization Strategic Advisory Group of Experts on Immunization. 2018 Assessment Report of the Global Vaccine Action Plan. 2018. https://apps.who.int/iris/bitstream/handle/10665/276967/WHO-IVB-18.11-eng.pdf?ua=1 [accessed August 20, 2019].

[5] MacDonald N, Mohsni E, Al-Mazrou Y, Andrus JK, Arora N, Elden S, et al. Global Vaccine Action Plan Review and Lessons Learned I: Recommendations for the Next Decade. Vaccine 2020;38:5364–71. 10.1016/j.vaccine.2020.05.003. PMC7342005 32563607

[6] Mantel C, McCabe A, Malvolti S. Developing a Global Immunization Strategy 2021-2030. Meeting of the Strategic Advisory Group of Experts (SAGE) on Immunization, Geneva, Switzerland. October 2017. https://www.who.int/immunization/sage/meetings/2017/october/3_16102017Mantel_GVAP_SAGEOct2017.pdf?ua=1 [accessed August 19, 2019].

[7] Daugherty M.A., Hinman A.R., Cochi S.L., Garon J.R., Rodewald L.E., Nowak G. (2019). The Global Vaccine Action Plan - insights into its utility, application, and ways to strengthen future plans. Vaccine.

[8] Lydon P. Global Immunization after 2020. Meeting of the Strategic Advisory Group of Experts (SAGE) on Immunization, Geneva, Switzerland. October 2018. https://www.who.int/immunization/sage/meetings/2018/october/SAGE_october_2018_GVAP_Lydon.pdf [accessed August 19, 2019].

[9] Gale N.K., Heath G., Cameron E., Rashid S., Redwood S. (2013). Using the framework method for the analysis of qualitative data in multi-disciplinary health research. BMC Med Res Methodol.

[10] World Health Organization. Global Immunization Vision and Strategy 2006-2015. 2005. http://whqlibdoc.who.int/hq/2005/WHO_IVB_05.05.pdf?ua=1 [accessed August 21, 2019].

[11] Declaration on “Universal Access to Immunization as a Cornerstone for Health and Development in Africa”. Addis Ababa, Ethiopia. 2016. https://www.afro.who.int/sites/default/files/2017-12/Addis%20Declaration%20on%20Immunization.pdf [accessed October 7, 2019].

[12] World Health Organization. Immunization Agenda 2030: A Global Strategy to Leave No One Behind. https://www.who.int/immunization/immunization_agenda_2030/en/ [accessed March 13, 2020].

[13] Cherian T, Hwang A, Mantel C, Veira C, Malvolti S, MacDonald N (2020). Global Vaccine Action Plan lessons learned III: Monitoring and evaluation/accountability framework.. Vaccine.

