# Supplementary material for: Global Vaccine Action Plan Lessons Learned II: Stakeholder Perspectives
Source: Vaccine. 2020 Jul 14;38(33):5372–8. doi: 10.1016/j.vaccine.2020.05.048 (PMC7342011; doi:10.1016/j.vaccine.2020.05.048)
Supplement: Supplementary data 1 [file mmc1.pdf]

**Global Vaccine Action Plan Lessons Learned II: Stakeholder Perspectives**


Angela Hwang[a, c*], Chantal Veira[b], Stefano Malvolti[c], Thomas Cherian[c], Noni MacDonald[d], Christoph Steffen[e], Ian Jones[f], Alan Hinman[b], Carsten Mantel[c]

[a] Angela Hwang Consulting, P.O. Box 6601, Albany, California, 94706. USA. angela@ahwang.net

[b] Task Force for Global Health, 330 West Ponce de Leon Ave., Decatur, Georgia, 30030. USA. cveira@taskforce.org, ahinman@taskforce.org

[c] MMGH Consulting GmbH, Kuerbergstrasse 1, 8049 Zurich, Switzerland. hwanga@mmglobalhealth.org, malvoltis@mmglobalhealth.org, mantelc@mmglobalhealth.org, cheriant@mmglobalhealth.org

[d] Dalhousie University, IWK Health Centre, Halifax, Canada. noni.macdonald@dal.ca

[e] Immunization, Vaccines and Biological Department, World Health Organization, Geneva, Switzerland. steffenc@who.int

[f] Jinja Publishing Ltd, Bishop's Stortford, United Kingdom. i.jones@jinjapublishing.co.uk

* Corresponding author


**SUPPLEMENTAL MATERIALS**


Appendix A GVAP Strategic Objectives ..... 2

Appendix B Interview Series 1 Questions ..... 3

Appendix C Survey 2 Questions ..... 4

Appendix D Survey 3 Questionnaire ..... 7

Appendix E Interview Series 2 Questions ..... 15

Appendix F Survey 3 Full Results and Comparison of Global vs. Regional and Country Perspectives ..... 17

Appendix G Thematic Analysis of Stakeholder Interviews ..... 20

## Appendix A GVAP Strategic Objectives

| | |
|---|---|
| **Strategic Objective 1** | All countries commit to immunization as a priority. |
| **Strategic Objective 2** | Individuals and communities understand the value of vaccines and demand immunization as both their right and responsibility. |
| **Strategic Objective 3** | The benefits of immunization are equitably extended to all people. |
| **Strategic Objective 4** | Strong immunization systems are an integral part of a well-functioning health system. |
| **Strategic Objective 5** | Immunization programs have sustainable access to predictable funding, quality supply and innovative technologies. |
| **Strategic Objective 6** | Country, regional, and global research and development (R&D) innovations maximize the benefits of immunization. |

**Appendix B Interview Series 1 Questions**

1. Was the GVAP development process adequate to the goals and time available?
2. Were the resources involved in the process sufficient and appropriate?
3. Was regional and country involvement sufficient?
4. Were representatives of the different functional areas (policy setting, implementation, R&D, advocacy, communications, funding, etc.) sufficiently involved?
5. Were each of these functional areas dealt with sufficiently and appropriately?
6. Was the goal and objective setting process balanced and strategic?
7. Did it contribute to create accountability?
8. Has the resulting plan been actionable and with sufficient implementation focus?
9. What will be a likely scenario/situation of global immunization by 2030?
10. Is there a need for a GVAP 2.0 while the GVAP 1.0 has not yet delivered on all its goals?
11. Should GVAP 1.0 merely be extended to achieve its goals? Or with modified goals?
12. Top-down vs. bottom-up (country involvement)? Should the development of GVAP 2.0 be mainly a regional rather than global process?
13. What would this mean in terms of financing support?
14. How should the target setting be done? Can region-specific goals and objectives be incorporated into a Global Vaccine and Immunization Framework?
15. What importance should be given to Advocacy and Communications? With which objectives?
16. Should overall funding needs be fully addressed ahead of the start of the plan?
17. How could currently existing financing streams for immunisation be incorporated (Gavi, Coalition for Epidemic Preparedness Innovations (CEPI), etc.)?
18. How can integration with the broader health/Sustainable Development Goal agenda and with other major global programs be ensured (e.g. Global Fund, etc.)?
19. Is there agreement with the timeline (initiating the process in 2017 to arrive at a fully developed plan (based on strong regional components) in time for approval at WHA 2020?

**Appendix C Survey 2 Questions**

1. **Why do you think a new global immunization strategy post-GVAP is needed?**

   - A new strategy is needed because immunization has always been led by global strategies whether to eradicate smallpox in the 1960s up to the GVAP in 2010
   - A new strategy is needed because immunization has lost visibility within the broader health agenda narrative and a global strategy can help re-position immunization within UHC and SDG3
   - A new strategy is needed because most of the goals set out for the Decade of Vaccines will not have been achieved and we need a strategy that emphasizes that GVAP goals remain relevant post-2020 and the global partnership needs to accelerate progress to achieve them
   - A new strategy is needed because the global immunization landscape has significantly changed since the development of GVAP with new and emerging issues that need to be tackled post-2020
   - A new strategy is needed because donors need a global strategy to help with their investment decisions or replenishment efforts

2. **What would be the overarching objective of developing a new global immunization strategy post-GVAP?**

   - The objective of a new strategy would be to re-iterate the value of investing in vaccines and immunization as a key intervention for PHC and UHC
   - The objective of a new strategy would be to prioritize and coordinate future immunization efforts for collective action
   - The objective of a new strategy would be to clarify roles & responsibilities of partners and stakeholders at all levels – global, regional, country
   - The objective of a new strategy would be to describe new and game changing actions and interventions to deliver on immunization outcomes
   - The objective of a new strategy would be to support advocacy and fundraising purposes

3. **Who do you think will be the main target audience of such a new post-2020 immunization strategy?**

   - Global policy makers
   - Regional advisory groups
   - Countries
   - International donors / funding mechanisms
   - Bilateral development agencies

- UN agencies
- NGO community
- Private sector
- Other not listed

**4. If a new post-GVAP immunization strategy is developed, what should be its main focus?**

- The main thrust of the strategy would be to describe why investing in immunization is imperative to reach UHC and SDG3
- The main thrust of the strategy would be to describe what the global immunization community needs to be prioritizing post-2020
- The main thrust of the strategy would be to articulate a framework for how to drive change in countries (the how to implement change)

**5. Many new and emerging issues have been put forward by various partners in the past years. How would you rank the following in terms of their importance for post-2020?**

- Reducing pockets of unimmunized located sub-nationally to raise coverage and equity
- Reducing the immunization gaps in middle income countries
- Strengthening immunization delivery platforms through the life course
- Addressing re-emerging and new disease threats including those that may be impacted by climate change
- Building national ownership for sustainable immunization programmes
- Developing new approaches to immunization for the growing number of fragile states
- Sustaining immunization gains with population growth, rising urbanization & increased migration
- Stepping up efforts on VPD disease surveillance in the context of health security
- Soft landing for countries that will be transitioning out of GPEI, Gavi, Global Fund…
- Creating a sustainable demand for vaccines and reducing pools of susceptibles due to vaccine hesitancy

**6. If a new post-GVAP immunization strategy is developed, how do you think it should it be organized?**

- The new strategy should be organized around remaining GVAP goals that need to be achieved post-2020
- The new strategy should be organized around main components of the immunization systems to strengthen over the next decade (ex: data quality, supply chain, sustainability, new vaccine introduction, service delivery…)

- The new strategy should be organized around new and emerging issues in global immunization that need to be addressed (those listed in Q5 above and others)
- The new strategy should be organized by countries in different income groups in order to differentiate the issues to tackle between low, middle and high income countries (ex: coverage/equity in LICs; access to vaccines at affordable prices in MICs; vaccine hesitancy in HIC)
- The new strategy should be organized by level of responsibility to define who is responsible to do what at global, regional and country level

7. **Do you think a new post-GVAP strategy will require that specific technical sub-strategies to be developed as inputs?**

**Note**: For Survey 1 Questions, see Daugherty MA, Hinman AR, Cochi SL, Garon JR, Rodewald LE, Nowak G, et al. *The Global Vaccine Action Plan - insights into its utility, application, and ways to strengthen future plans*. Vaccine. 2019;37:4928-36. DOI: 10.1016/j.vaccine.2019.07.042

## Appendix D Survey 3 Questionnaire

As we approach the end of Decade of Vaccines, the immunization community is actively engaged in understanding the successes and challenges of the Global Vaccine Action Plan (GVAP) as a crucial input to developing a post-2020 immunization strategy. To this end, previous surveys have collected feedback on how the GVAP was developed and on the utility of GVAP annual reports and recommendations.

This survey follows up to collect your views on how the GVAP was implemented, focusing specifically on the **Added Value of GVAP**. We will interpret your responses as personal perspectives, not necessarily representing your present or past institutions.

You will see several lists of key actions that were undertaken in conjunction with GVAP.
Please **score each action** in terms of how much it contributed to improving immunization systems as follows:

Impact on Improving Global Immunization
3 – important contribution
2 – moderate contribution
1 – slight contribution
0 – no contribution

Please score the impact of each action in **your own professional context**, whether national, regional, or global, and consider only the **impact attributable to GVAP.**

*For example, if the action significantly strengthens an immunization system, and that improvement would* ***not*** *have happened without GVAP, then it would be scored 3, for "important contribution". If the improvement would have happened even without GVAP, the action would be scored 0, for "no contribution".*

**1) Monitoring and Evaluation/Accountability Framework.** The following actions were undertaken at the global and regional levels to foster accountability for achieving GVAP goals.

**a) Indicators and Targets.**

The GVAP Monitoring and Evaluation/Accountability Framework reinforced or enhanced existing global targets and established a wide range of new indicators and targets for issues such as financing, integration, and research and development. Since 2017, progress against key indicators has been available online at the GVAP Indicators Portal.

*How much did this build accountability for improving global immunization?*

**b) Regional Annual Reports**.

Since 2016, WHO regions have published annual progress reports of their regional vaccine action plans developed in conjunction with the GVAP Secretariat Report. These reports have been presented in regional committee (RC) meetings each year.

*How much did this help build accountability for improving global immunization?*

**c) Global Annual Reports.**
The GVAP Secretariat describes global progress toward GVAP targets each year in a comprehensive Secretariat Report.

*How much did this help build accountability for improving global immunization?*

**d) Independent monitoring and review.**
The Strategic Advisory Group of Experts (SAGE) reviews the Secretariat report and issues a concise ***Assessment Report*** that highlights key issues and **recommends actions** to accelerate progress.

*How much did this help build accountability for improving global immunization?*

**e) Independent oversight**.
As called-for by the World Health Assembly (WHA), the WHO Executive Board (EB), and the WHA review progress on an annual basis to foster accountability at the highest levels.

*How much did this help build accountability for improving global immunization?*

**f) Link with the Global Strategy for Women's, Children's and Adolescents' Health.** SAGE Assessment Reports are shared with the United Nations Secretary-General's Independent Accountability Panel for Every Woman, Every Child, Every Adolescent to inform their annual progress reviews.

*How much did this help build accountability for improving global immunization?*

**g) Multi-partner engagement.**
The GVAP was developed under the auspices of 5 major global health institutions (WHO, UNICEF, Gavi, the Bill and Melinda Gates Foundation, and NIAID (USA), and these organizations engaged actively in the monitoring process, including serving as the secretariat for preparing annual reports.

*How much did this help build accountability for improving global immunization?*

**h) Are there any additional actions relating to GVAP that *have contributed to* improving global accountability for reaching global immunization goals? Please describe and score their impact.**

**2) GVAP Strategic Objective 1: All countries commit to immunization as a priority.**
The following actions were undertaken at global and regional levels to build political will for immunization.

**a) Guiding Principles**.
Six principles were adopted to guide the elaboration of GVAP: 1) Country ownership, 2) Shared responsibility and partnership, 3) Equity, 4) Integration, 5) Sustainability, and 6) Innovation.

*How much did this contribute to building political will for improving global immunization?*

**b) Global Goals**.
The GVAP set forth 5 Goals:

- Achieve a world free of poliomyelitis
- Meet global and regional elimination targets
- Meet vaccination coverage targets in every region, country and community
- Develop and introduce new and improved vaccines and technologies
- Exceed the Millennium Development Goal 4 target for reducing child mortality

*How much did this contribute to building political will for improving global immunization?*

**c) World Health Assembly (WHA) Actions**.
In 2012, the WHA endorsed the GVAP, and in 2013 it adopted the GVAP Monitoring and Evaluation Framework. It adopted two resolutions in response to the annual reports: one on greater vaccine affordability in 2015 and the Midterm Review resolution in 2017.

*How much did this contribute to building political will for improving global immunization?*

**d) Regional Vaccine Action Plans.**
By 2016, all the WHO regions had adopted regional vaccine action plans aligned with the GVAP. These plans include robust monitoring and evaluation (M&E) frameworks that contribute to global GVAP M&E.

*How much did this contribute to building political will for improving global immunization?*

**e) Addis Declaration on Immunization**.
At the 28th African Union (AU) Summit in 2017, Heads of State from across Africa endorsed the Addis Declaration on Immunization (ADI), committing to advance universal access to immunization across Africa. This was accompanied by a roadmap for its implementation.

*How much did this contribute to building political will for improving global immunization?*

**f) National Immunization Technical Advisory Groups** (NITAGs).
GVAP called for an increase in the number of countries with functioning NITAGs and Assessment Reports have recommended that NITAGs contribute to monitoring the implementation of national vaccine action plans.

*How much did this contribute to building political will for improving global immunization?*

**g) Economic Evidence in support of immunization**.
The Decade of Vaccines Economics (DOVE) project has generated economic evidence on the value of vaccines, including estimates of the cost of illness, return-on-investment, and the cost of financing vaccine programs.

*How much did this contribute to building political will for improving global immunization?*

**h) Political will for strengthening immunization programs.**

*Overall, did GVAP contribute to building political will for immunization program strengthening in your context?*

**i) Are there any additional actions relating to GVAP that have contributed to political will or for otherwise improving global immunization? Please describe and score their impact.**

**3) GVAP Strategic Objective 2: Individuals and communities understand the value of vaccines and demand immunization as both their right and responsibility.**
The following actions were undertaken at the global and regional levels to build demand for immunization.

**a) Immunization advocacy and communications.**
GVAP messages have been disseminated through World Immunization Weeks, #VaccinesWork, and other immunization-related media

*How much did this contribute to building and maintaining demand for improving global immunization?*

**b) GVAP-related scientific articles.**
Publications addressing GVAP have included special issues of Health Affairs and Vaccine. See additional examples at LINK.

*How much did this contribute to building and maintaining demand for improving global immunization?*

**c) CSO engagement.**
GVAP highlighted the role that CSOs play in ensuring that leadership and accountability are in place at all levels (local, national, regional and global). The GVAP Secretariat has engaged CSO representatives in monitoring the progress of GVAP.

*How much did this contribute to improving global immunization?*

**d) Vaccine confidence and demand**.
GVAP called for monitoring trends in the level of confidence in vaccination. In response, indicators of vaccine demand and hesitancy have been added to the Joint Reporting Form, which countries use to report immunization data to WHO and UNICEF. The SAGE Working Group on Vaccine Hesitancy was launched to understand and help address hesitancy; its outputs have been published on the WHO website and in a special issue of Vaccine.

*How much did this contribute to improving global immunization?*

**e) Visibility for immunization.**

*Overall, did GVAP help to increase visibility or improve communication and advocacy for immunization?*

**f) Are there any additional actions relating to GVAP that have contributed to building and maintaining demand for immunization? Please describe and score their impact.**

**4) Strategic Objective 3: The benefits of immunization are equitably extended to all people.**
The following actions were undertaken at the global and regional levels to improve equity in immunization.

**a) Immunization coverage targets**.
GVAP reaffirmed the coverage targets set by the Global Immunization Vision and Strategy, calling for achieving at least 90% national DTP3 coverage and 80% DTP3 coverage in every district in all member states by 2015. For the first time, GVAP also set targets for a) reducing inequity in coverage between wealth quintiles, b) reducing dropout rates, and c) sustaining high coverage for three or more consecutive years.

*How much did this contribute to improving equity in global immunization?*

**b) Subnational data**.
GVAP reviews have contributed to a greater appreciation of the need for subnational data to evaluate progress in immunization and to efforts to collect, share, and use subnational data. As of 2018, 141 member states have reported subnational immunization data.

*How much did this contribute to improving equity in global immunization?*

**c) New vaccine introduction target**.
GVAP called for at least 90 low and lower-middle income countries to introduce one or more new or underutilized vaccines by 2015, and for all such countries to introduce one or more new or underutilized vaccines by 2020.

*How much did this contribute to improving equity in global immunization?*

**d) Focus on fragile countries and vulnerable populations**.
GVAP Assessment Reports have highlighted the challenges presented by conflict and crisis. They have called for partner coordination and targeted approaches to reach children consistently missed, especially in countries with low vaccination rates and in populations displaced by conflict.

*How much did this contribute to improving equity in global immunization?*

**e) Measles and rubella/congenital rubella syndrome (CRS) elimination**.
GVAP Assessment reports have noted the importance of high and equitable coverage in achieving elimination, highlighted the challenges of achieving measles and rubella/CRS elimination targets and called for additional resources, strengthening immunization systems and improving case-based surveillance.

*How much did this contribute to improving equity in global immunization?*

**f) Maternal and neonatal tetanus elimination (MNTE).**
GVAP Assessment reports have highlighted missed targets for MNTE, noted that maternal and neonatal tetanus were a very visible manifestation of inequity, and called for concerted efforts to achieve elimination by 2020.

*How much did this contribute to improving equity in global immunization?*

**g) Equity in immunization.**

*Overall, how much did GVAP contribute to improving equity in immunization in your context?*

**h) Are there any additional actions relating to GVAP that have contributed to equity in immunization? Please describe and score their impact.**

**5) Strategic Objective 4: Strong immunization systems are an integral part of a well-functioning health system.**

The following actions were undertaken at the global and reginal levels to strengthen immunization systems and foster greater coordination between immunization and other programs.

**a) Global Routine Immunization Strategies and Practices (GRISP)**.
To help operationalize the GVAP, the GRISP provides a comprehensive framework of strategies and practices for routine immunization intended to help realize the full benefits of immunization.

*How much did this help to foster coordination and improve global immunization?*

**b) Data quality targets and tools**.
GVAP established a target of all countries having high quality immunization coverage data by 2020, as determined by the WUENIC Grade of Confidence, and highlighted the need to improve data quality in multiple Assessment Reports. The Data Quality Review Toolkit was published in 2017 to provide guidance to countries in conducting annual reviews of data quality.

*How much did this help to foster coordination and strengthen global immunization?*

**c) Joint Reporting Form (JRF) and data quality workshops**.
As a result of data quality concerns raised by the first GVAP report, JRF workshops are now being held in all regions to improve the quality of the reported data. Regional workshops for data quality are also being held.

*How much did this help to foster coordination and strengthen global immunization?*

**d) Integration into wider health systems**.
An indicator assessing health system integration (including immunization) was approved by SAGE in 2017. In addition, WHO has developed the Missed Opportunities for Vaccination Strategy (MOVS) to increase coverage and promote synergy between programs.

*How much did this help to foster coordination and strengthen global immunization?*

**e) Translation of GVAP into the national plans through the cMYP process.**
The operationalization of the GVAP at the country level was meant to be through the updating of the national comprehensive multi-year plans (cMYP). Guidance was provided on how to align national cMYPs to the GVAP.

*How effective was this in improving global immunization?*

**e) Immunization Systems and Integration**

*Overall, did GVAP help to strengthen immunization systems and foster integration into wider health systems in your context?*

**f) Are there any additional actions relating to GVAP that have contributed to strengthen immunization and foster coordination? Please describe and score their impact.**

**6) Strategic Objective 5: Immunization programs have sustainable access to predictable funding, quality supply and innovative technologies.**
The following actions were undertaken at the global and regional levels to address these issues.

**a) Immunization financing indicator**.
GVAP called for an increasing trend in country financing of national immunization programs. Assessment Reports have recommended that countries improve the tracking and reporting of immunization expenditures.

*How much did this contribute to improving global immunization?*

**b) Vaccine quality indicator**.
GVAP monitored the percentage of doses of vaccine used worldwide that are of assured quality.
*How much did this contribute to improving the quality of vaccine supply and to global immunization?*

**c) Vaccine supply**.
GVAP monitoring highlighted the issue of vaccine stockouts and contributed to greater attention to the problem of vaccine supply. The MI4A project (Market Information for Access to Vaccines) is now gathering market intelligence on vaccine supply and demand to address affordability and shortage issues for self-funding and self-procuring countries.

*How much did this contribute to improving access to vaccine supply and to global immunization?*

**d) Vaccine pricing**.
At the 2015 World Health Assembly, countries raised their concerns about vaccine prices and adopted a landmark resolution calling for price transparency and greater affordability. This created momentum for the V3P platform, which facilitates the appropriate comparison of price information and to provide countries with accurate, reliable and useful data on vaccine product, price and procurement.

*How much did this contribute to the affordability of vaccines and to improving global immunization?*

**e) Access to predictable funding and quality supply.**

*Overall, how much did GVAP improve predictable funding and access to supply in your context?*

**f) Are there any additional actions relating to GVAP that *have contributed* to improve financing and supply in immunization? Please describe and score their impact.**

**7) Strategic Objective 6: Country, regional, and global research and development (R&D) innovations maximize the benefits of immunization.**

The following actions were undertaken at the global and regional levels to accelerate high-impact R&D in vaccines and immunization.

**a) R&D indicators**. GVAP established new indicators and targets calling for:

1. Licensure and launch of vaccines against one or more major currently non-vaccine preventable diseases
2. Licensure and launch of at least one platform delivery technology
3. Progress towards development of HIV, TB, and malaria vaccines
4. Progress towards a universal influenza vaccine
5. Progress towards institutional and technical capacity to carry out vaccine clinical trials
6. Vaccines that have either been re-licensed or licensed for use in a controlled-temperature chain
7. Vaccine delivery technologies receiving WHO prequalification

Assessment reports have called for improving research capacity in low- and middle-income countries and making more use of implementation and operational research to improve immunization system performance.

*How much did this help to accelerate R&D to improve global immunization?*

**b) Memorandum of Understanding (MOU) on Enhanced Research-Focused Institutional Collaboration related to the Global Vaccine Action Plan**.
In 2013, leaders of the WHO, the US National Institute of Allergy and Infectious Diseases, and the Bill and Melinda Gates Foundation Global Health Program signed a MOU to strengthen and develop research-focused institutional cooperation in relation to the Decade of Vaccines.

*How much did this help to accelerate R&D to improve global immunization?*

**c) Global Vaccines and Immunization Research Forum (GVIRF).**
The GVIRF is held every 2 years to assess progress in the GVAP R&D agenda, identify opportunities and challenges in meeting GVAP goals, and promote partnerships in vaccine research.

*How much did this help to accelerate R&D to improve global immunization?*

**e) Vaccine Research and Development.**

*Overall, how much did GVAP accelerate vaccines and immunization research and development in your context?*

**f) Are there any additional actions relating to GVAP that have contributed to improving research and development for immunization? Please describe and score their impact.**

## Appendix E Interview Series 2 Questions

1. Please describe your involvement to date with the Decade of Vaccines Collaboration (DOVC), the Global Vaccine Action Plan (GVAP), and, if applicable, your Regional Vaccine Action Plan (RVAP).
2. What went well in the DOVC? Specifically, with respect to structure, process, partnership and collaboration?
3. What went poorly in the DOVC? Specifically, with respect to structure, process, partnership and collaboration?
4. Was there enough regional and country involvement? If not, please explain. ... or what could have been done better?
5. In your view, what were the strengths and weaknesses of the GVAP partnership and collaboration?
6. How have the GVAP and RVAPs influenced your organization's immunization goals, priorities, and strategies?
7. Have the resulting regional and country plans been actionable and with a sufficient implementation focus?
8. Did immunization activities in countries benefit from 'GVAP branding'? If so, please provide examples.
9. Has GVAP helped to build demand for immunization? If so, please provide examples.
10. Have GVAP and 'your' RVAP helped mobilize funds for immunization in your country/region/organization and if so, how?
11. Each year the GVAP Assessment Reports and regional progress reports make recommendations for accelerating progress. Which recommendations have been the most relevant and useful to your organization?
12. In your view, was the GVAP (and RVAP) monitoring / evaluation and accountability framework fit for purpose? Did the indicators, targets, and annual review process contribute to accountability and trigger corrective action in countries?

13. Each year, progress under GVAP is discussed at the World Health Assembly and each RVAP is discussed at relevant Regional Committee meetings. Do you perceive these discussions as useful? Have they helped to build political will for immunization?
14. What was the greatest contribution of GVAP to R&D for immunization? What could have been done better?
15. Looking back to the call for the Decade of Vaccines in 2010, how has the immunization landscape changed since then? Has progress accelerated, kept pace, or slowed? How have the adoption of the GVAP in 2012 and the RVAPs contributed to this change?
16. Given the changes that have occurred in the immunization landscape since 2010, what are the most important aspects of the GVAP to retain going forward? Similarly, going forward, what are the most important aspects to revise? In terms of the most useful partnership?
17. Do you have any additional thoughts to share, on any of the topics we've discussed today?

## Appendix F Survey 3 Full Results and Comparison of Global vs. Regional and Country Perspectives

**Perceived GVAP contribution to improving global immunization**: score distribution and average score for each of the 36 survey items (all respondents combined), sorted by average score

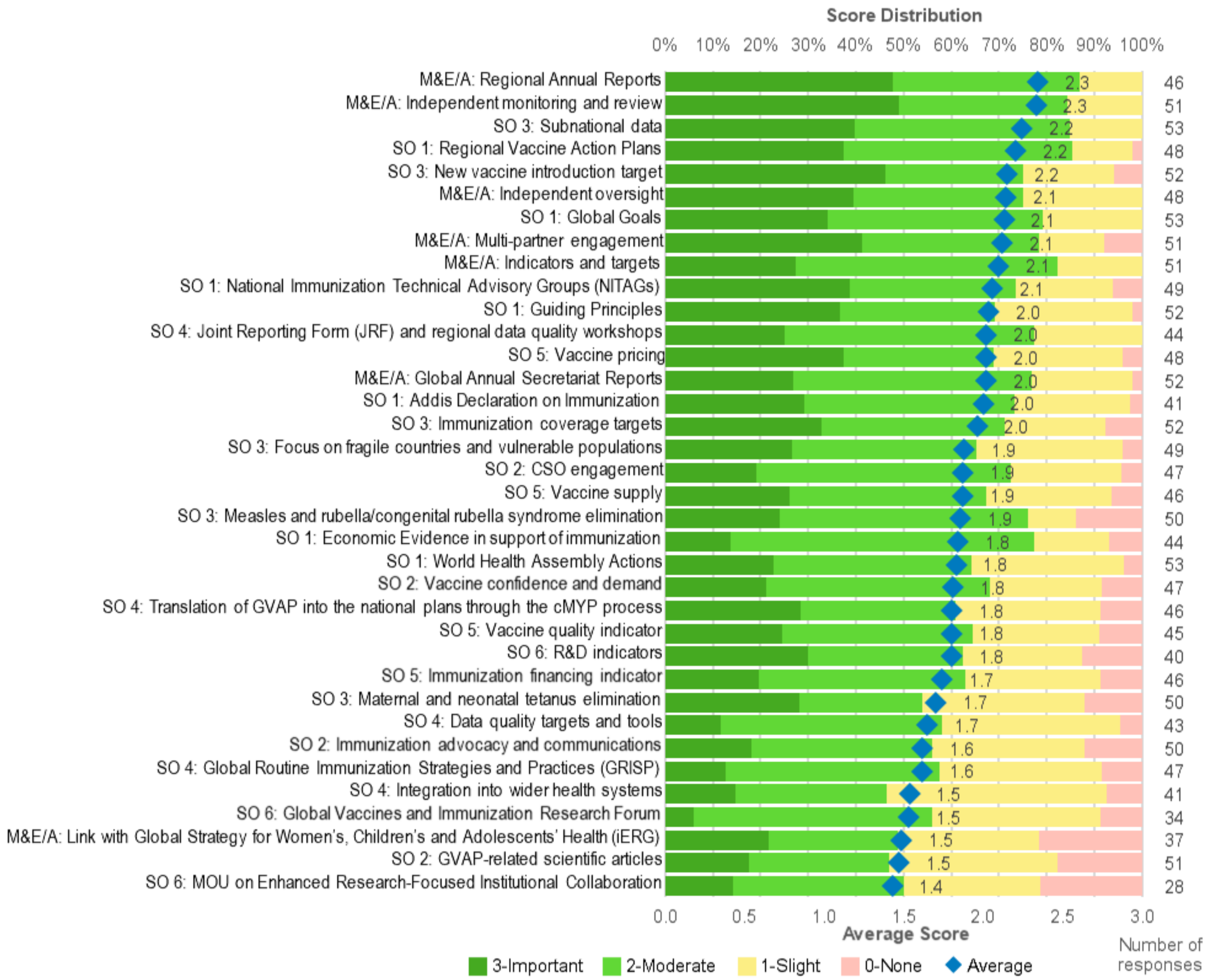

**Perceived GVAP contribution to improving global immunization**: average score for each of the 36 survey items, by respondent perspective (global or regional/country) (N=56)

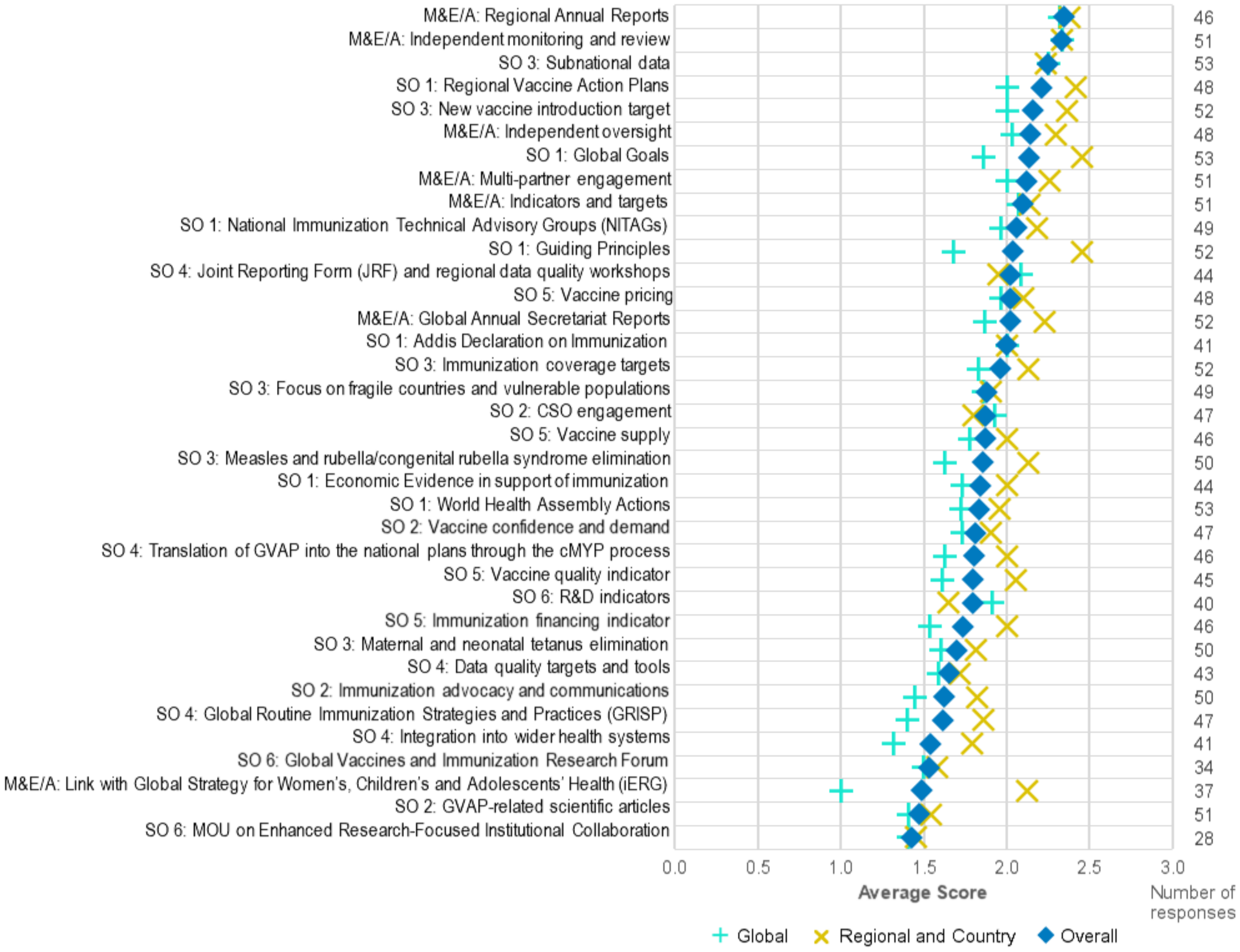

**GVAP Contribution to achieving Strategic Objectives**: average score for each of the 36 survey items, by respondent perspective (global or regional/country) (N=56)

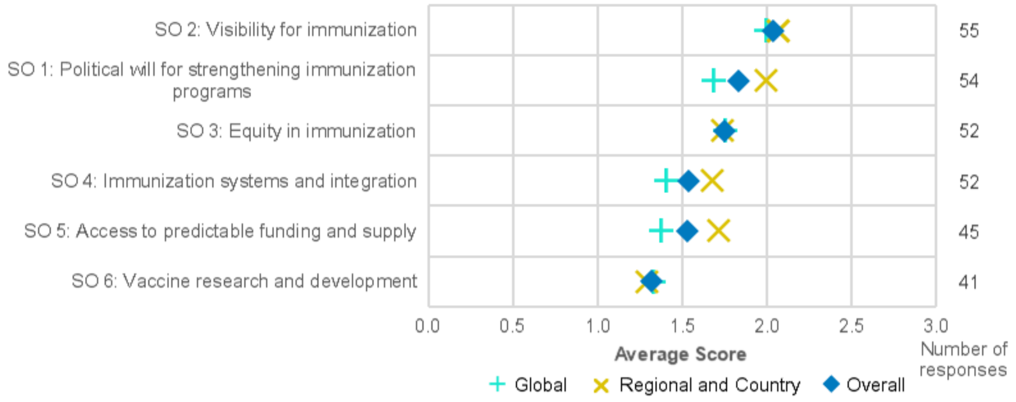

## Appendix G Thematic Analysis of Stakeholder Interviews

**Preparation of GVAP**. GVAP was developed through a consultative process. While the consultations were unprecedented in scope and many said that they were robust and well-conducted, some commented that country and regional involvement was insufficient and that timelines were too rushed for appropriate feedback. Others noted that the overall process lacked clarity. In the final writing phase, the country voice and connections with partners were lost, resulting in a strategy seen as "top-down" by stakeholders from all perspectives. This also contributed to misalignments around some aspects of the plan, such as resource mobilization, implementation, and stakeholder roles. Ultimately, the process was robust enough to develop the content of the strategy, but not sufficient to engender country ownership.

**Country ownership**. The lack of widespread country ownership was seen as an important shortcoming of GVAP. While some countries, for example in the Americas, have taken the lead in implementing GVAP, in other countries GVAP was associated closely with WHO and UNICEF and did not make much of a difference in national activities. The lack of country ownership was attributed to the development process, in which GVAP was seen as developed by a small set of agencies; misalignment between country priorities and GVAP goals; and a misalignment between ambitious GVAP targets and the limited resources available to tackle them. It was also attributed to "unrealistic" targets adopted in GVAP, although it should be noted that these targets did not originate with GVAP but had been endorsed at previous WHAs by country representatives.

**GVAP partnerships**. GVAP contributed to partnerships for immunization by engaging additional stakeholders, for example including the US National Institute for Allergies and Infectious Diseases (NIAID) in the Leadership Council and strengthening the role of civil society organizations (CSOs). In addition to NIAID, the Leadership Council included executives from the Bill & Melinda Gates Foundation, Gavi, UNICEF, and WHO. While active during the preparation of GVAP, during the decade their role diminished. WHO was seen as the driving force for GVAP and considered an appropriate lead for the strategy. At the country level UNICEF and WHO were seen as leading actors in GVAP implementation. Gavi, which was seen as the main implementing partner for GVAP, was less deeply involved in the preparation of GVAP and did not adopt GVAP targets that they considered unrealistic. The main weakness of the GVAP partnerships was incomplete ownership: partners did not subscribe to GVAP in its

entirety but set their own priorities. As a result, some issues were not sufficiently supported during the decade.

**GVAP Strategy**. The GVAP strategy was seen as comprehensive and sound, and many elements of it have been implemented. Compared to its predecessor, the Global Immunization Vision and Strategy 2006-2015 (GIVS) [10], it was more visible, drew in more stakeholders and placed more emphasis on advocacy and communications and research and development. That said, some noted that prioritization of interventions would have been useful. Views varied on the level of ambition in GVAP: some spoke to the need for aspirational goals, noting that "if you put the target high, you'll do better." Others recommended realistic goals, noting that aspirational goals undermine implementation and accountability. One person commented that eradication goals distract from system strengthening. Multiple respondents recommended a balance between aspiration and realism.

**Advocacy**. Many noted that GVAP was used as an advocacy tool, serving as a call to action, highlighting the importance of immunization across many audiences, including Ministers of Health, and contributing to political will for immunization. Others were less positive, noting that advocacy for GVAP and immunization was limited, weak, or declined after GVAP endorsement. Many immunization stakeholders at country level appeared to have limited awareness and understanding of GVAP.

**Resources**. Experience regarding resource mobilization varied from country to country, with some respondents noting that GVAP had helped to mobilize domestic resources and others saying that it had not. Among partner organizations, some said GVAP had helped increase resources dedicated to immunization while others said that GVAP did not shape their budgets. Responses relating to resources seemed especially subject to inaccuracies: in multiple cases respondents said that GVAP had led to increases in funding by specific organizations—statements that were directly contradicted in other interviews by representatives of those organizations.

On the whole, there was a mismatch between the ambitious goals adopted by GVAP and the resources dedicated to achieving them. This was seen as a barrier to country ownership and accountability for achieving GVAP targets, especially for middle-income countries not eligible for Gavi support. In addition, the pledge made by Bill Gates at the launch of the Decade of Vaccines to contribute USD 10 billion to immunization created unrealistic expectations.

**Implementation**. After GVAP endorsement, regional vaccine action plans (RVAPs) were updated or developed in alignment with GVAP for each WHO region. The RVAPs translated GVAP to the regional context, making them more action-oriented and adapting goals to regional situations. Countries contributed to development of the RVAPs, and in general the RVAPs were seen as more relevant to countries than GVAP. Country plans, which were the main driver of GVAP implementation, were in many cases updated or developed to align with the relevant RVAP. In fewer cases, the RVAP was said to have little influence on a particular country plan. Partners at the global level aligned their strategies with GVAP. Respondents noted multiple challenges leading to incomplete GVAP implementation, including the delays in aligning the RVAPs to GVAP, a lack of clarity on the resources required for implementation, insufficient funding to achieve the ambitious goals adopted by GVAP, lack of country ownership, and lack of buy-in by implementing partners. Similar challenges hampered implementation of the recommendations made in the annual reporting process.

**Monitoring and Evaluation/Accountability (M&E/A).** The GVAP M&E/A framework included indicators and targets corresponding to each goal and strategic priority established in GVAP and an annual review process consisting of reporting, independent review and recommendations by the WHO Strategic Advisory Group of Experts on Immunization (SAGE), and discussions at annual World Health Assembly (WHA) meetings. Similar processes were followed at the regional level to monitor progress on RVAPs.

Global M&E/A was considered a highlight of GVAP. Data collection for new indicators such as stockouts and vaccine hesitancy helped to draw attention to these issues. The annual SAGE reports were praised, with respondents noting that they were widely read among global stakeholders, used for advocacy, and helped to increase visibility for immunization. Recommendations made by SAGE helped to highlight important issues such as data quality and the need to serve vulnerable populations. Discussions at WHAs and WHA side meetings brought these issues to the attention of Ministers of Health, created opportunities to discuss challenges such as vaccine affordability for middle-income countries, and may have contributed to political will for immunization. Concerns regarding the global M&E/A process included too many indicators, too great a focus on missed targets and insufficient recognition of progress, and vague or impractical recommendations.

M&E/A was also seen as an important element of the RVAPs, although there was a greater diversity of opinion as expected given regional diversities. The M&E/A process supported regional offices in country interactions. Annual RVAP progress reports served to highlight emerging issues and were reviewed at regional immunization technical advisory group meetings and EPI managers meetings. Discussions at Regional Committee meetings were important opportunities to underscore the importance of immunization and to draw attention to countries that were falling behind. Some respondents differed, noting that the regional reports garnered little attention at Regional Committee meetings.

Country level respondents gave widely differing feedback on the M&E/A process. Some noted that it built awareness of progress, but others noted that GVAP-related M&E/A was not helpful. Multiple respondents expressed concerns about data quality. Several country respondents were unaware of the annual GVAP reports and their recommendations, however one country respondent said that the GVAP recommendations helped to garner support from national decision makers. Some noted that WHA sessions on GVAP were a good reminder of the importance of immunization and could be used to move the agenda forward, others said that these discussions were political and not linked to any actions in the country, and some were unaware of the WHA discussions of immunization.

Overall, the M&E/A process was seen as a necessary and important element of GVAP. It was a useful process and contributed to accountability for achieving GVAP goals, but was unable to ensure widespread, full accountability for meeting GVAP targets.

**Accountability.** Lack of accountability for achieving GVAP targets was an important issue for regional and global respondents. In contrast, it was mentioned only twice in country interviews—those two respondents also noted a lack of accountability. Accountability was difficult to achieve due to aspirational targets and because stakeholders were not aligned on some targets, such as measles elimination. It was also difficult to achieve because GVAP lacked a unified management structure directing implementation and controlling resources: the GVAP governance structure was seen as weak, with "no teeth." Some noted greater accountability to RVAP targets and to the "10 Commitments" of the Addis Declaration on Immunization, which was launched to increase political will for immunization in Africa. Some respondents questioned why GVAP did not hold partners accountable, focusing its assessments on country progress while partners operated according to their own priorities and plans.

**GVAP value-add.** The greatest observed benefit from GVAP lay in aligning stakeholders around a common agenda with a common language, which contributed to partnership, collaboration, advocacy, and, in some instances, fundraising. It widened the pool of stakeholders, for example engaging the private sector, and highlighted important issues such as coverage, equity, vaccination after infancy, data quality, and vaccine supply and stockouts. It strengthened NITAGs and aligned global stakeholders around a research and development agenda, provided a platform for information exchange and brought researchers and implementers together, and may have prevented some companies from pulling out prematurely out of vaccine development projects.

Significant progress was made during the decade across many GVAP goals, and some respondents noted that GVAP helped improve immunization performance. However, the majority were unsure about the extent to which GVAP had contributed to this progress, or noted that this progress would have happened without GVAP. Attribution of benefits to GVAP is inherently difficult because the strategy reinforced existing goals and initiatives and was implemented in the context of many overlapping interventions and of existing programs and strategies supporting immunization, such as those of WHO, UNICEF and Gavi, the Vaccine Alliance.

**Need for a post-2020 global immunization strategy.** There was consensus among respondents that an updated immunization strategy is needed to sustain the benefits of immunization, complete the unfinished business of GVAP, and address emerging challenges. In addition to advancing existing priorities such as coverage and equity, elimination and eradication targets, data quality, vaccine uptake, and operational research, it should improve demand for immunization and address vaccine hesitancy, improve access to sufficient supplies of affordable vaccines for middle-income countries, promote the integration of immunization with other health services, and address the needs of people affected by fragility, conflict, and vulnerability.

**Success factors**. Respondents made many suggestions for improving the next global immunization strategy. These suggestions included:

- **Country ownership** is will be essential for success. For strong country ownership, national stakeholders need to play greater roles in developing the new strategy. The strategy should emphasize implementation and implementing partners such as civil society organizations should be involved in its development.

- **Global strategy**. In addition to the needs of low-income countries, respondents commented that the new strategy should address the challenges facing high- and middle-income countries, including sustaining progress in countries with declining support from Gavi and other funders. It should foster cross-country communication and learning. It should focus on a small number of priority actions and goals, and balance disease-specific goals, including eradication and elimination targets, with systems strengthening. It should have a greater focus on enabling factors such as trust, governance, accountability, and domestic resources.
- **Living strategy**. The strategy should be able to adapt to changing contexts and new challenges. It should be more agile and dynamic, and one respondent recommended a planned, mid-course reconfiguration. It should feature innovation as an important tool for improving delivery, addressing hesitancy, and driving improvements where progress has stalled.
- **Strong and expanded partnership**. All stakeholders should be able to agree on shared, core principles and there should be good alignment with the Gavi strategy. Respondents called for partnering beyond immunization programs to facilitate implementation and integration: the new strategy should align with related initiatives and concerns such as global health security, disease-specific initiatives, primary health care and universal health coverage. Partnerships can also help mobilize resources and build political will and accountability: the new strategy should engage with Ministers of Finance, regional development banks, parliaments and the African Union, civil society organizations, multinationals and the private sector. The strategy should specify roles and responsibilities for this wider set of stakeholders.
- **Financial plan.** Clarity on resource requirements and funding streams is needed from the outset. This should be supported by economic analysis showing the return on investment for immunization, and costing at the country level, which was seen as more useful than global costing. The financial plan should emphasize domestic resources for immunization and seek to widen the pool of donors. In light of anticipated declines in development assistance, the new plan should emphasize the benefits of improved coordination and efficiency in resource utilization.
- **"Bottom-up" goalsetting**. Goals need to be relevant to different country settings and achievable. Deeper country engagement in goalsetting was seen as essential to country ownership and accountability, and perspectives varied on how this could be achieved. Many supported a bottom-up approach, in which countries set their own goals and targets, which then inform regional targets that in turn drive the global framework. Some respondents differed,

suggesting instead a regionally driven approach in which objectives and targets are set at the regional level, or a mixed approach in which implementation objectives are defined at the country level but visionary goals are defined at the global level. Some respondents also noted that existing goals should be retained.

- **Country-tailored implementation**. Global recommendations and technical assistance should be adapted to the country context. Maturity models and solution archetypes can facilitate implementation of country-tailored approaches.
- **Governance and Accountability**. Respondents commented that the governance mechanism should have country and regional buy-in and engagement. Some recommended looking beyond the WHA and enlisting political bodies, such as the African and European Unions, in governance. Partners should also be held accountable for their role in the new strategy.
- **M&E/A**. There should be fewer indicators in the new measurement framework. It should monitor progress in implementation as well as outcomes, including implementation of recommendations issued over the course of the decade. It should explore ways to measure political will and decision making for immunization. Evaluations should be independent, recognize progress even when targets are missed and explore the root causes for missed targets. Regional offices would benefit from support to monitor progress.
- **Advocacy** will be an important component of the new strategy, to highlight the importance of immunization, build political will and country ownership, and address vaccine hesitancy. It should promote domestic investments to sustain progress in immunization and be linked with advocacy for related causes such as primary health care.
